# Dr. Leroy E. Jones

**Published:** 1902-03

**Authors:** 


Dr. Leroy E. Jones died at Buffalo, January 5, 1902, aged 81 years.

